# Association of follow-up neutrophil-lymphocyte ratio with postoperative pneumonia in aneurysmal subarachnoid hemorrhage patients after endovascular treatment: a retrospective analysis

**DOI:** 10.3389/fmed.2025.1493761

**Published:** 2025-07-01

**Authors:** Xinyue Huang, Xutang Jiang, Wen Gao, Liming Guo, Xiumei Guo, Hanlin Zheng, Zhigang Pan, Qingxin Lin, Shuni Zheng, Chuhan Ke, Weipeng Hu, Zhen Qi, Feng Zheng

**Affiliations:** ^1^Department of Neurosurgery, The Second Affiliated Hospital, Fujian Medical University, Quanzhou, China; ^2^Department of Neurology, The Second Affiliated Hospital, Fujian Medical University, Quanzhou, China; ^3^Division of Public Management, The Second Affiliated Hospital, Fujian Medical University, Quanzhou, China; ^4^Division of Neurosurgery, Jinjiang Municipal Hospital (Shanghai Sixth People's Hospital Fujian Campus), Quanzhou, China

**Keywords:** neutrophil-lymphocyte ratio, aneurysmal subarachnoid hemorrhage, postoperative pneumonia, endovascular treatment, predict

## Abstract

**Introduction:**

Research on the relationship between dynamic neutrophil-to-lymphocyte ratio (NLR)—measured at different time points around endovascular treatment—and the occurrence of postoperative pneumonia (POP) in patients with aneurysmal subarachnoid hemorrhage (aSAH) is still relatively scarce. This study aims to explore the association between dynamic NLR and the incidence of POP in patients with aSAH undergoing endovascular therapy.

**Methods:**

This study included patients with aSAH who underwent endovascular treatment between January 2019 and April 2023. The follow-up endpoint was the presence of POP at 30 days postoperatively. Logistic regression analysis was conducted using POP as the dependent variable. The NLR was calculated at admission (NLR1), 24 h after endovascular treatment (NLR2), and 3–7 days after endovascular treatment (NLR3). Four prediction models were constructed: Model 1 (variables with *p* < 0.05, except for the NLR), Model 2 (Model 1 plus NLR1), Model 3 (Model 1 plus NLR2), and Model 4 (Model 1 plus NLR3).

**Results:**

Among the 154 patients with aSAH, POP occurred in 101 (65.6%) patients. Higher NLRs at admission [odds ratio (OR) = 1.08; 95% confidence interval (CI): 1.02, 1.16; *p* = 0.019], 24 h postoperatively (OR = 1.14; 95% CI = 1.05, 1.25; *p* = 0.005) and 3–7 days postoperatively (OR = 1.17; 95% CI = 1.02, 1.38; *p* = 0.04) were associated with the occurrence of POP.

**Conclusion:**

Follow-up NLR may be associated with POP in patients with aSAH treated endovascularly. Elevated NLR at admission, 24 h postoperatively, and 3–7 days postoperatively correlated with a high risk for POP.

## 1 Introduction

Aneurysmal subarachnoid hemorrhage (aSAH) is a cerebrovascular disorder with potentially devastating consequences and a mortality rate that ranges from 8.3 to 66.7% (1, 2). Treatment typically involves surgical and endovascular procedures (3, 4), and endovascular procedures have emerged as a predominant trend recently (5). The yearly incidence rate of aSAH is 7.9 cases per 100,000 people (6, 7), and the incidence of serious complications of postoperative pneumonia (POP) occurring within 30 days ranges from 13 to 37% (8–10). Pneumonia during hospitalization significantly increases mortality among patients who experienced ischemic stroke, cerebral hemorrhage, or aSAH (11, 12). Therefore, the identification of reliable predictors of pneumonia is crucial for screening high-risk patients who require active surveillance and appropriate interventions to improve their outcomes and prognosis.

The neutrophil-to-lymphocyte ratio (NLR) is a widely recognized indicator of systemic inflammation and infection and has been extensively examined as a predictor of various diseases (13, 14). Compared to traditional inflammatory markers, NLR has a superior predictive value (15, 16). Previous research has identified that NLR is associated with cerebral ischemia (17, 18) and brain tumors (19, 20). Furthermore, NLR has shown potential as a prognostic indicator for POP in patients with aSAH (21). However, research on the relationship between dynamic NLR—measured at different time points before and after endovascular treatment—and the occurrence of POP in patients with aSAH is still relatively scarce.

Therefore, this study aimed to explore the association between dynamic inflammatory markers (specifically NLR measured at various time points before and after endovascular treatment) and the occurrence of POP in patients with aSAH undergoing endovascular therapy.

## 2 Materials and methods

### 2.1 Patient population

This is a retrospective, single-center study conducted at the Second Affiliated Hospital of Fujian Medical University, which included patients with aSAH who underwent endovascular treatment between January 2019 and April 2023. The data were accessed for research purposes on October 1, 2023. The patients were treated in accordance with the current clinical protocols in a real-world environment (22). This study is based on the current version of the ‘Declaration of Helsinki,’ and all research processes are conducted in accordance with the relevant guidelines and regulations. The research in question received the necessary approval from the Institutional Review Board of the Second Affiliated Hospital of Fujian Medical University, China (Ethical approval no. 315/2023). As the study is retrospective in nature, the requirement for informed consent was waived by the review board.

The inclusion criteria were as follows: (1) SAH caused by intracranial aneurysm confirmed through computed tomography angiography (CTA) or digital subtraction angiography (DSA); (2) admission within 7 days of emergence of symptom; and (3) patients treated endovascularly.

The following exclusion criteria were used: (1) individuals with connective or autoimmune disease, uremia, hematologic disease, malignancy, cirrhosis, or chronic pulmonary disease; (2) patients admitted with a diagnosis of pneumonia; (3) patients who had undergone surgery 1 month before admission or had an acute inflammatory or infectious disease within the last 3 months; (4) patients receiving antibiotics, systemic glucocorticoids, immunosuppressants, or immunotherapy; and (5) patients who were measured for NLR less than two times.

### 2.2 Data collection

Patient demographic characteristics and vascular risk factors, encompassing age, sex, hypertension, diabetes, hyperlipidemia, coronary disease, current smoking and alcohol history, previous history of hemorrhagic and ischemic stroke, treatment received (coiling or stent-assisted coiling), aneurysm location, multiple occurrences and sizes determined via CTA or DSA, and routine blood tests, were acquired from the electronic medical record system. Blood specimens were procured, and NLR was calculated at three time points: (1) at admission (neutrophil 1, lymphocyte 1, and NLR1); (2) 24 h after endovascular therapy (neutrophil 2, lymphocyte 2, and NLR2); and (3) 3–7 days after endovascular treatment (neutrophil 3, lymphocyte 3, and NLR3) (23, 24). If multiple values were available, the earliest neutrophil and lymphocyte counts were chosen to reduce reverse causation bias. To infer any causality, only N/L values collected before the diagnosis of pneumonia should be included in the analysis. NLR was established as the absolute neutrophil count divided by lymphocyte count, which was obtained from complete blood counts. The severity of aSAH was classified according to the grading system proposed by the World Federation of Neurosurgical Societies (WFNS) (25). The relationship between aSAH severity and cerebral vasospasm was classified using the modified Fisher (mFisher) scale (26). The patient’s medical data were evaluated to derive the preliminary WFNS classification and mFisher grade.

### 2.3 Outcome assessment

The participants were categorized into two groups based on the occurrence of POP. According to the revised criteria by the Centers for Disease Control and Prevention (CDC) (27), the patients were diagnosed with POP if they contracted a lower respiratory tract infection within 30 days postoperatively, with at least one chest radiograph confirming the diagnostic alteration. Patients with a preadmission diagnosis of pneumonia were ruled out.

### 2.4 Statistical analysis

The dataset was analyzed using the R software (version 4.2.2). Continuous variables with normal distribution are expressed as the mean ± SD, whereas other variables are represented as the median + interquartile range (IQR). Data were analyzed using the *t*-test and Mann–Whitney U test for continuous variables, the *χ*^2^ test, and Fisher’s exact test for categorical variables. Independent predictors of POP after aSAH were ascertained using univariate and multifactor logistic regression analyses. In the univariate logistic regression analysis, variables with *p* values < 0.1 were incorporated into the multifactor logistic regression model. In the final multivariate model, variables with *p* values < 0.05 were considered significantly correlated. Subsequently, inverse stepwise selection was used to ascertain parsimonious models limited to the most pertinent variables, and variables in the model were selected for significance at a level of 0.05. The following four prediction models were constructed: (I) Model 1, with statistically significant variables (*p* < 0.05) except for NLR; (II) Model 2 (Model 1 augmented by NLR1); Model 3 (Model 1 augmented by NLR2); and Model 4 (Model 1 augmented by NLR3). Odds ratios (ORs) and 95% confidence intervals (CIs) were computed. The diagnostic accuracy of these factors in predicting POP was evaluated using receiver operating characteristic (ROC) curves and the area under the curve (AUC). Delong test was utilized to assess the significance of the differences in AUC values of the models. Due to the high percentage of missing data for some exposures (missing data < 20%), we performed a secondary analysis by repeating the primary analysis after multiple inputs of missing data. We assumed that the data were missing at random (dependent on the values of other non-missing variables rather than the values of the missing variables). We performed multiple imputations by chained equations (MICE) using R software.

## 3 Results

**Figure 1:**
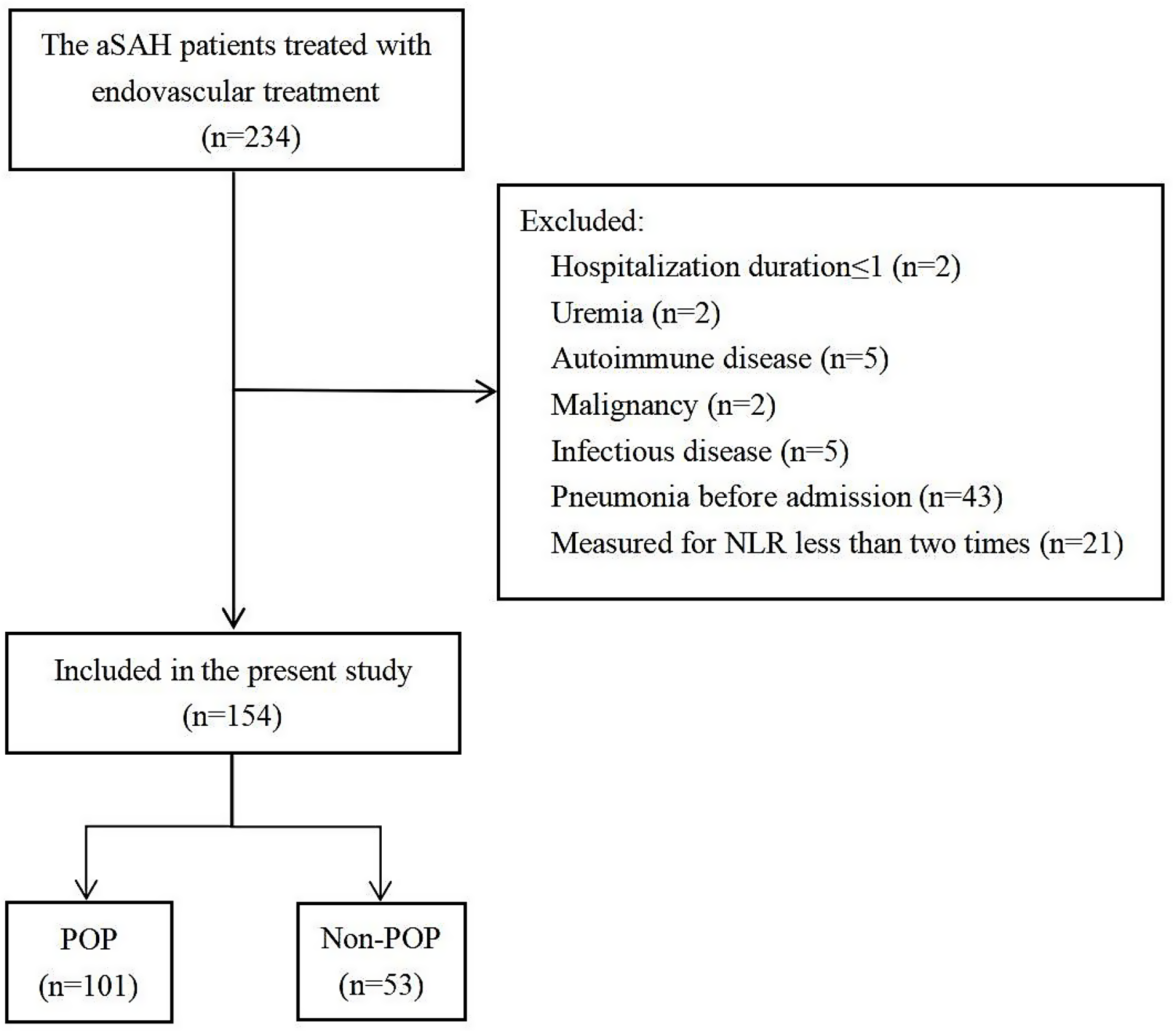
Flow chart of the patients included in the analysis. aSAH, aneurysmal subarachnoid hemorrhage; POP, postoperative pneumonia.

A cohort of 235 individuals with aSAH received endovascular treatment, of whom 154 met the inclusion criteria (Figure 1) and were divided into the POP (*n* = 101) and non-POP (*n* = 53) groups. The average age of the patients was 57.51 ± 12.62 years, and 64.3% (99/154) were women. The baseline characteristics of the participants are presented in Table 1.

**Table 1:** Comparison of baseline characteristics in patients with aSAH who were treated endovascularly according to the occurrence of POP.

Variables	POP (*n* = 101)	Non-POP (*n* = 53)	*p*-value
Age, years, mean ± SD	54.43 ± 11.41	54.43 ± 11.41	0.022^a^
Age >62, *n* (%)	44 (44%)	12 (23%)	0.010^b^
Sex, female, *n* (%)	37 (37%)	18 (34%)	0.742^b^
Hypertension, *n* (%)	61 (60%)	31 (58%)	0.819^b^
Diabetes, *n* (%)	6 (5.9%)	2 (3.8%)	0.715^c^
Hyperlipidemia, *n* (%)	6 (5.9%)	3 (5.7%)	>0.999^c^
Coronary heart disease, *n* (%)	3 (3.0%)	2 (3.8%)	>0.999^c^
Smoking history, *n* (%)	7 (6.9%)	3 (5.7%)	>0.999^c^
Drinking history, *n* (%)	7 (6.9%)	2 (3.8%)	0.719^c^
Ischemic stroke, *n* (%)	1 (1.0%)	2 (3.8%)	0.272^c^
Hemorrhagic stroke, *n* (%)	2 (2.0%)	1 (1.9%)	>0.999^c^
Multiple aneurysms	21 (21%)	9 (17%)	0.571^b^
Ruptured aneurysm size (mm)	5.00 (3.98, 6.63)	5.00 (3.60, 6.00)	0.572^d^
Location			0.070^b^
Anterior circulation aneurysm	88 (87%)	51 (96%)	
Posterior circulation aneurysm	13 (13%)	2 (3.8%)	
GCS at admission			0.007^b^
3–8	20 (20%)	2 (3.8%)	
9–12	24 (24%)	9 (17%)	
13–15	57 (56%)	42 (79%)	
WFNS grade	2.00 [1.00, 4.00]	1.00 [1.00, 2.00]	<0.001^d^
mFisher grade	4.00 [2.00, 5.00]	1.00 [1.00, 2.00]	<0.001^d^
Treatment			0.332^b^
coiling	25 (25%)	17 (32%)	
coiling & stent	76 (75%)	36 (68%)	
EVD, *n* (%)	5 (5%)	2 (3.8%)	0.739^c^
Lumbar drainage, *n* (%)	5 (5.9%)	1 (1.9%)	0.251^c^
Hospitalization duration, days median [IQR]	19 (15, 26)	15 (14, 17)	<0.001^d^
Laboratory parameters			
Neutrophil1, 109/L, median [IQR]	11.4 (9.3, 15.0)	8.2 (6.4, 11.0)	<0.001^d^
Lymphocyte1, 109/L, median [IQR]	1.25 (0.92, 2.00)	1.30 (0.98, 1.99)	0.928^d^
NLR1, median [IQR]	9 (6, 14)	6 (4, 12)	0.030^d^
Neutrophil2, 109/L, median [IQR]	8.66 (6.91, 11.71)	9.3 (7.2, 11.3)	0.024^d^
Lymphocyte2, 109/L, median [IQR]	1.26 (0.96, 1.64)	1.15 (0.92, 1.78)	0.142^d^
NLR2, median [IQR]	6.8 (4.6, 9.4)	6.7 (4.7, 10.4)	0.019^d^
Neutrophil3, 109/L, median [IQR]	11.1 (9.0, 13.8)	7.93 (6.45, 8.59)	0.004^d^
Lymphocyte3, 109/L, median [IQR]	1.03 (0.73, 1.29)	1.41 (1.20, 1.62)	0.008^d^
NLR3, median [IQR]	11.2 (7.9, 16.9)	5.5 (4.5, 7.1)	<0.001^d^

SD, standard deviation; GCS, Glasgow Coma Scale; WFNS, World Federation of Neurosurgical Societies; mFisher, modified Fisher; EVD, external ventricular drainage; POP, postoperative pneumonia; NLR, neutrophil-to-lymphocyte ratio; IQR, interquartile range; *p < 0.05.

a *T* test.

b Pearson’s Chi-squared test.

c Fisher’s exact test.

d Wilcoxon rank sum test; Neutrophil1, Lymphocyte1, and NLR1 were tested at admission; Neutrophil2, Lymphocyte2, and NLR2 were tested within 24 h after endovascular treatment; Neutrophil3, Lymphocyte3, and NLR3 were tested 3–7 days after endovascular treatment.

Compared to the non-POP group, the POP group was characterized by older age (*p* = 0.022), lower Glasgow Coma Scale (GCS) scores at admission (*p* = 0.007), higher WFNS grades (*p* < 0.001), higher Fisher grades (*p* < 0.001), and increased ventilator use (*p* < 0.002). Additionally, NLR1 (*p* = 0.03), NLR2 (*p* = 0.019), and NLR3 (*p* = 0.001) increased significantly in the POP group compared to those in the non-POP group (Table 1).

**Figure 2:**
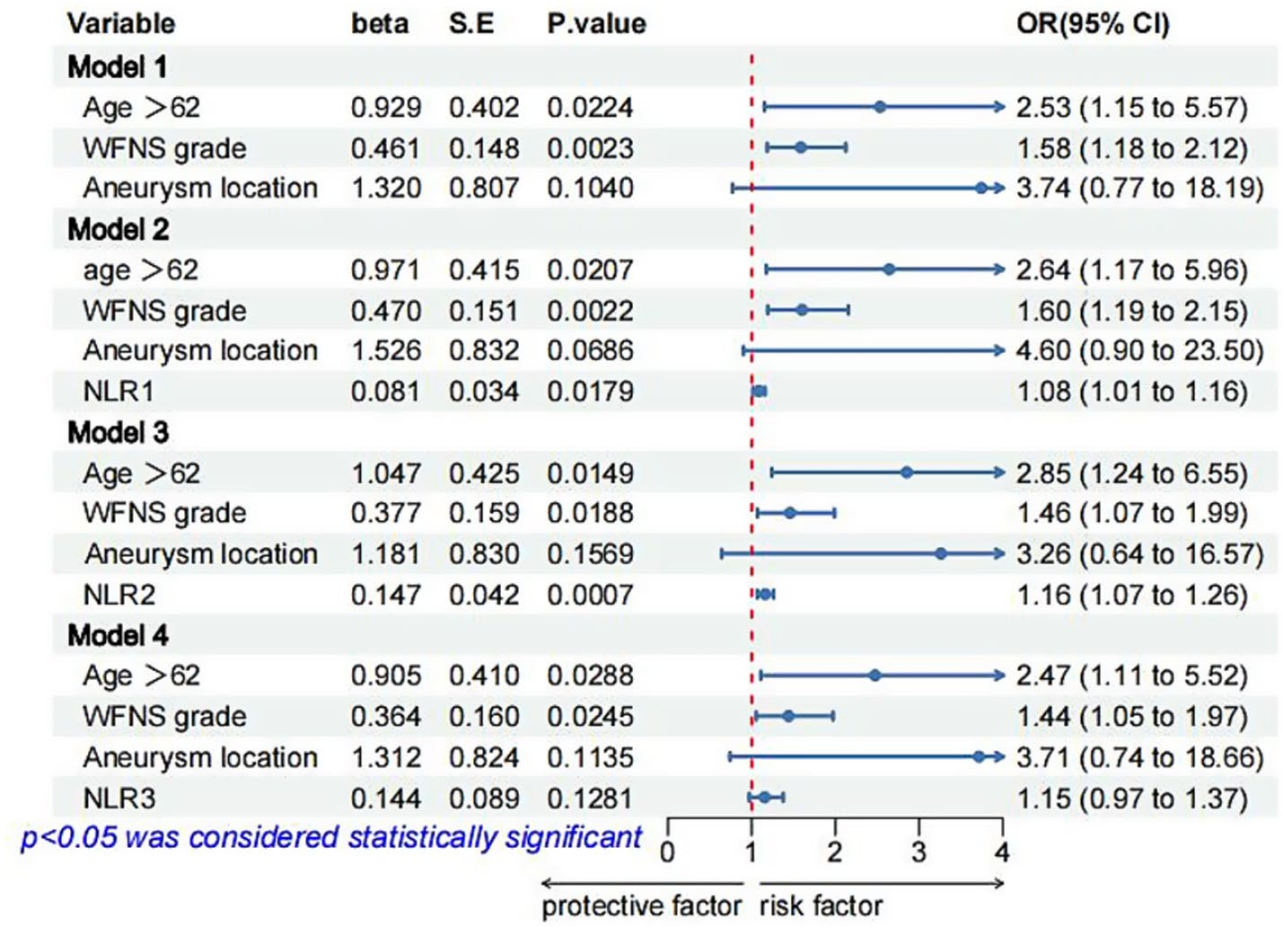
Logistic regression models for POP in patients with aSAH after multiple imputation of missing data. OR, odds ratio; CI, confidence interval; SE, standard error; POP, postoperative pneumonia; NA, not available; NLR1, NLR2, and NLR3 were measured at the periods reported in Table 1.

**Figure 3:**
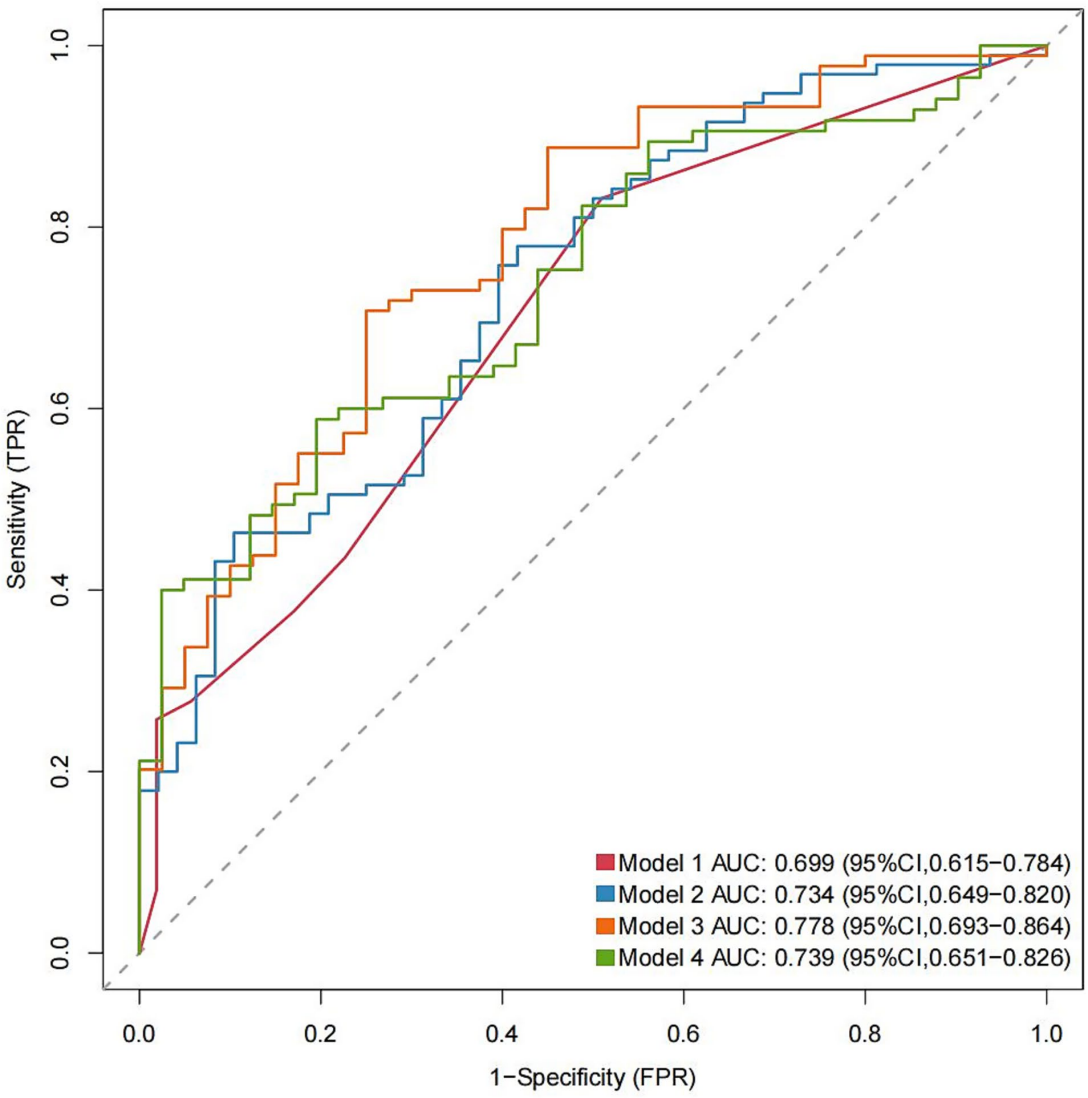
AUC values for each model with different NLR measurements (Models 1–4). AUC, area under the curve. The significant variables age > 62 years and WFNS grade were selected to construct Model 1; age > 62 years, WFNS grade, and NLR1 for Model 2; age > 62 years, WFNS grade, and NLR2 for Model 3; and age > 62 years, WFNS grade, and NLR3 for Model 4. The parameters were measured at the specified periods reported in Table 1.

**Figure 4:**
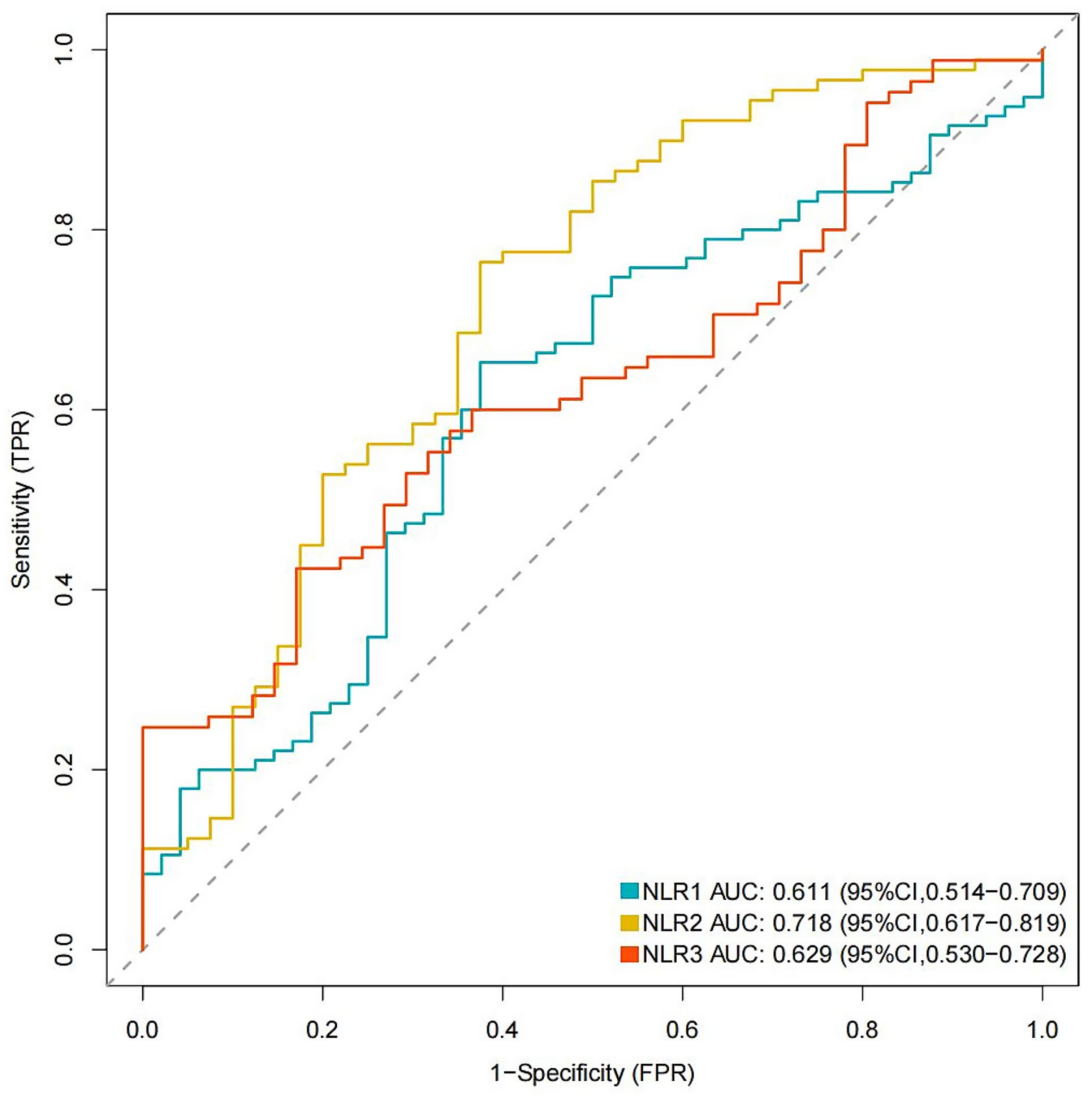
ROC curves of NLR and POP in different time points. ROC, receiver operating characteristic; POP, postoperative pneumonia; NLR1, NLR2, and NLR3 were measured at the periods reported in Table 1.

Multivariate logistic regression analysis showed that a higher NLR at admission (OR = 1.08; 95% CI 1.02, 1.16; *p* = 0.019), 24 h postoperatively (OR = 1.14; 95% CI 1.05, 1.25; *p* = 0.005) and 3–7 days postoperatively (OR = 1.17; 95% CI 1.02, 1.38; *p* = 0.04) were independently linked to the occurrence of POP. By ROC curve analysis, the optimal cut-off value for the age-maximizing Youden index is 62. Age > 62 years and higher WFNS grades were associated with the occurrence of POP in all four models (Table 2). Imputation of missing data did not substantially change the estimated association (Figure 2). The AUC was utilized to calculate the diagnostic accuracy of the four models, with values of 0.699, 0.734, 0.778, and 0.739 for models 1–4, respectively (Figure 3). Delong test showed that among these models, the difference in AUC values between model 3 and model 1 was statistically significant (*p* = 0.049), indicating model 3 has better predictive performance. ROC curves of NLR and POP in different time points were shown in Figure 4. Details of sensitivity, specificity, positive predictive value, negative predictive value and the cutoff in each time point were shown in Table 3.

**Table 2:** Logistic regression models for POP in patients with aSAH.

Variables	Model 1		Model 2		Model 3		Model 4	
	OR [95% CI]	*p*	OR [95% CI]	*p*	OR [95% CI]	*p*	OR [95% CI]	*p*
Age >62	2.53 [1.17, 5.74]	0.021*	2.58 [1.14, 6.19]	0.028*	2.64 [1.184, 5.883]	0.013*	2.88 [1.20, 7.43]	0.022*
WFNS grade	1.59 [1.20, 2.16]	0.002*	1.63 [1.22, 2.27]	0.002*	1.49 [1.07, 2.15]	0.025*	1.44 [1.03, 2.06]	0.038*
Aneurysm location	3.74 [0.92, 25.4]	0.10	3.46 [0.79, 24.5]	0.14	2.59 [0.57, 18.6]	0.3	2.89 [0.64, 20.9]	0.2
NLR1	NA	NA	1.08 [1.02, 1.16]	0.019*	NA	NA	NA	NA
NLR2	NA	NA	NA	NA	1.14 [1.05, 1.25]	0.005*	NA	NA
NLR3	NA	NA	NA	NA	NA	NA	1.17 [1.02, 1.38]	0.040

OR, odds ratio; CI, confidence interval; POP, postoperative pneumonia; NA, not available; *p < 0.05; NLR1, NLR2, and NLR3 were measured at the periods reported in Table 1.

**Table 3:** AUC in predicting POP in different NLR measurements.

	Sensitivity, %	Specificity, %	Positive predictive value, %	Negative predictive value, %	Cutoff point
NLR1	65.3	62.5	77.5	47.6	6.944
NLR2	76.4	62.5	81.9	54.3	7.566
NLR3	42.4	82.9	83.7	41.0	7.562

AUC, area under the curve; POP, postoperative pneumonia; NLR1, NLR2, and NLR3 were measured at the periods reported in Table 1.

## 4 Discussion

In the present study, NLR at admission, 24 h postoperatively and 3–7 days postoperatively, age > 62 years, and WFNS grade were were found to be associated with POP in patients with aSAH who were treated endovascularly. Our study contributes to the early prediction of POP and timely intervention in patients with aSAH after endovascular treatment.

As a factor that increases the mortality rate of patients, especially those with aSAH, POP has received considerable attention from researchers and clinicians (11, 12). NLR has been shown to be a prognostic indicator of POP in individuals with aSAH (21). In our study, the results showed that NLR was also associated with pneumonia in individuals with aSAH who received endovascular treatment, which is consistent with prior research (21). Our investigation also revealed that age > 62 years and higher WFNS grade were associated with POP in individuals with aSAH. Previous studies have also shown that age, as a continuous variable, is closely related to pneumonia (28, 29). In the present study, the ROC curve identified 62 years as the optimal age cutoff point. Consequently, subsequent analyses employed this threshold for dichotomizing age variables. The augmented susceptibility of infection in individuals aged > 62 years may be due to low immunity and poor physiological status. The increased risk of infection in patients with higher WFNS grades indicates that aSAH severity may be correlated with POP. Therefore, intensive care is recommended as early as possible for patients with high WFNS scores, especially for older individuals. The results of these secondary analyses were largely similar to the primary analyses after multiple imputations of missing data, indicating that missing data had little effect on the results.

The current study explores the correlation between POP and NLR measured at different time points in patients with endovascularly treated aSAH. As the inflammatory response may develop in the few days after the onset of aSAH, NLR values must be obtained at various time points to explore the predictive value of NLR for POP. Upon incorporation of NLR1, NLR2, and NLR3 into our POP prediction models, the predictive capabilities of Models 2, 3, and 4 were higher than that of Model 1, with the highest predictive power detected in Model 3, which may be due to dynamic fluctuations in NLR levels over time. Therefore, the risk of POP may be assessed dynamically using NLR measurements at different time points. These results suggest that neuroinflammation may represent a potential and amenable objective for early identification and treatment of POP in patients with aSAH who receive endovascular treatment.

Our study found that NLR at admission, 24 h postoperatively, and 3–7 days postoperatively were associated with POP in patients with aSAH. The plausible relationship between NLR and POP can be explained by several factors. aSAH causes neutrophil activation and lymphopenia, leading to elevated NLR and inducing inflammatory responses and deregulation of the immune system to promote POP occurrence. Neurological impairment prompted by the inflammatory response and liberation of cytokines from immune cells has been discovered to elicit the generation of anti-inflammatory signals. These signals inhibit cytokine production, culminating in infection control and prevention of disease advancement (30). However, it should be noted that a prolonged inflammatory response can eventually lead to the exhaustion of the immune system (31). The resultant effect is a reduction in systemic immunity and concomitant suppression of systemic cellular immune responses. This syndrome is commonly referred to as stroke-induced immunosuppression syndrome and is characterized by a rapid decline in peripheral blood lymphocyte subpopulations (9, 32). As a result, patients become more susceptible to stroke-associated pneumonia. Various investigations have also discovered that NLR has the potential as a dependable forecaster of pneumonia among patients with ischemic stroke and aSAH, which may indicate a shared mechanism of excessive inflammation and immune imbalance that occurs after these acute cerebrovascular diseases (17). Therefore, regulation of the inflammatory and immune status by targeting particular subsets of neutrophils or lymphocytes may present a promising therapeutic objective.

This study had some limitations. First, this was a retrospective single-center analysis with a relatively small sample size, which limited the model construction to integrate NLR values from multiple time points to observe the impact of NLR at each specific time point on the model’s predictive capacity. Future studies with large sample sizes are necessary to further explore this direction. Second, serologic markers were not measured continuously for 30 days after aSAH, and understanding the prognostic value of these markers for POP in patients with aSAH treated endovascularly within a longer period is thus difficult. Further studies should focus on this topic. Third, due to the lack of clinical data, further analysis of the correlation between POP and other markers of inflammation, such as C-reactive protein in serum and procalcitonin, cannot be achieved in the present study. Future studies should be focused on this issue. Fouth, due to the relatively small sample size, a multivariate generalized mixed model cannot be achieved in the present study. Future studies including large sample size are warranted to account for repeated measures with this model. Fifth, the timing of pneumonia was not reported in this study, which is a very relevant data. Further studies are needed to explore the classification based on the timing of pneumonia onset. Sixth, the development of delayed cerebral ischemia, hydrocephalus, rebleeding and other complications may increase the risk of pneumonia if they preceded the diagnosis of pneumonia, which were not included in the present models. Further attention should be paid to this issue in future studies. Last, the present study was focused on the prediction of POP with follow-up NLR in patients with aSAH treated endovascularly. Whether other infections may also be predicted with follow-up NLR cannot be determined in the present analysis. Further attention is warranted to be paid to this.

In conclusion, follow-up NLR has been shown to be associated with POP in patients with aSAH who are treated endovascularly. Elevated NLR at admission, 24 h postoperatively, and 3–7 days postoperatively correlated with a high risk for POP. To validate our observations, future prospective multicenter investigations with larger and more diverse patient cohorts are necessary.

## Data Availability

The data analyzed in this study is subject to the following licenses/restrictions: The raw data supporting the conclusions of this article will be made available by the authors, without undue reservation. Requests to access these datasets should be directed to Feng Zheng, dr.feng.zheng@gmail.com.
